# Distinct Metabolomic Alterations Are Associated With Physical Function, Weight Loss, and Muscle Mass in Men With Cancer

**DOI:** 10.1002/jcsm.70183

**Published:** 2026-01-18

**Authors:** Lindsey J. Anderson, Lu Xia, Haiming Kerr, Marin Cabrera, Fabien Chu, Jaqueline Rose, Peter C. Wu, Atreya Dash, Sina A. Gharib, Jose M. Garcia

**Affiliations:** ^1^ Geriatric Research, Education and Clinical Center Veterans Affairs Puget Sound Health Care System Seattle Washington USA; ^2^ Department of Medicine, Division of Gerontology and Geriatric Medicine University of Washington Seattle Washington USA; ^3^ Department of Statistics and Probability Michigan State University East Lansing Michigan USA; ^4^ Department of Surgery University of Washington Seattle Washington USA; ^5^ Department of Surgery Veterans Affairs Puget Sound Health Care System Seattle Washington USA; ^6^ Department of Urology Veterans Affairs Puget Sound Health Care System Seattle Washington USA; ^7^ Department of Urology University of Washington Seattle Washington USA; ^8^ Division of Pulmonary, Critical Care and Sleep Medicine University of Washington Seattle Washington USA

**Keywords:** cancer cachexia, functional impairment, handgrip, metabolomics, skeletal muscle, stair climb

## Abstract

**Background:**

Treatments for cancer cachexia, defined as involuntary weight and muscle mass loss leading to significant functional impairment, remain unavailable partly due to insufficient improvement of clinically meaningful outcomes in current trials. By reflecting downstream effects of cellular function, metabolomics may identify mechanisms contributing to poor functional performance. Previous metabolomic studies in cancer cachexia have identified alterations in amino acid metabolism with weight loss or low muscularity; none have examined perturbations with poor physical function. We hypothesized that distinct metabolic signals in plasma and muscle are associated with weight loss, low muscle mass, and impaired function in cancer cachexia.

**Methods:**

We enrolled patients planning elective laparotomy for gastrointestinal or genitourinary cancer. Handgrip strength (HGS), stair climb power (SCP), and fasting plasma were collected within 2 weeks prior to surgery; rectus abdominis samples were obtained during surgery. Metabolomic perturbations associated with physical function (HGS, SCP), muscularity (lumbar cross‐sectional area ‘CSA’ from opportunistic CT), or weight loss (> 5% over previous 6 months) were examined in plasma and muscle. The Mann–Whitney U‐test compared metabolite abundance between weight‐losing and weight‐stable patients, while Spearman's correlation tested associations of abundance with CSA, HGS, or SCP. The ‘Globaltest’ method assessed pathway alterations with weight loss, CSA, HGS, or SCP; the Benjamini‐Hochberg adjustment was used to control for false discovery.

**Results:**

Patients (*N* = 72) were male, median age 65 [interquartile range: 59–70], with 57% genitourinary cancer. Plasma and skeletal muscle metabolomic data were collected (*N* = 64 and *N* = 68, respectively). Weight loss was associated with significantly altered microbial, amino acid/derivative, fatty acid/lipid, and caffeine‐related metabolism pathways in plasma (adjusted *p* < 0.1). Lower CSA was associated with significantly altered fatty acid/lipid, galactose, glycerophospholipid, and histidine metabolism and bile secretion pathways in skeletal muscle (adjusted *p* < 0.1). Worse HGS was nominally associated with altered plasma branched chain amino acid biosynthesis and altered skeletal muscle glutathione metabolism (unadjusted *p* ≤ 0.05), while worse SCP was nominally associated with altered skeletal muscle amino sugar/nucleotide sugar metabolism and phenylalanine, tyrosine, and tryptophan biosynthesis (unadjusted *p* ≤ 0.05).

**Conclusions:**

Significant metabolomic alterations in plasma and skeletal muscle characterized cancer‐related weight loss and reduced CSA, respectively. Nominal, function‐specific alterations were detected with worse HGS and SCP, which were distinct from those associated with weight loss or low CSA. Future larger studies may further characterize metabolomic profiles related to various functional outcomes and guide development of therapeutic targets to improve functional performance.

## 1 Introduction

Cancer cachexia is a complex metabolic syndrome characterized by loss of muscle mass with or without loss of fat mass, not reversed by nutritional supplementation, and leading to progressive functional impairment [1]. The host response to tumour burden can increase production of cytokines, decrease anabolic tone, and centrally suppress appetite, leading to hypermetabolism, weight loss, and reduced quality of life [2]. There is currently no approved treatment for cancer cachexia in the United States or Europe and one of the main barriers to its development is the lack of consensus definition for, and assessment of, functional impairment among other clinically‐relevant outcomes [3]. Some Phase II studies have reported significant improvements in functional measures such as stair climb power (SCP) [4], handgrip strength (HGS) [5, 6], or the short physical performance battery [7] in treatment groups compared to placebo. However, Phase III interventional trials seeking approval for this indication have failed to induce clinically meaningful improvements in physical function despite inducing significant increases in lean body mass [8, 9, 10, 11, 12].

The discrepancy between improvements in muscle mass and function in clinical trials highlights the need to further understand mechanistic alterations in skeletal muscle contributing to poor functional performance. High throughput ‘omics’ analyses are frequently used to characterize the spectrum of molecules present in a sample or cohort and identify altered biomarker or pathway profiles in disease settings [13]. By capturing the range of small molecules (metabolites) that represent the end process of cellular pathways, metabolomics reflects the ultimate downstream effects of perturbations to the genome, transcriptome, and proteome [14].

Metabolomic analyses in cancer cachexia to date have predominantly reported altered amino acid metabolism in the circulation or urine from weight‐losing patients compared to those with stable weight [15, 16, 17, 18, 19, 20]. Amino acid catabolism and impaired glucose metabolism in the circulation were also observed in cancer patients with low skeletal muscle index [21] and with muscle wasting [22] compared to those with normal skeletal muscle index or stable/increased muscle mass, respectively. These studies are limited by small sample size and/or focus on weight loss or muscularity alone.

Identification of metabolites and metabolic pathways related to poor functional performance may reveal potential targets for future therapeutic strategies. The purpose of this study was to characterize metabolomic signatures in plasma and skeletal muscle in patients with cancer that are associated with cachexia‐related outcomes such as poor physical function as measured by HGS or SCP, low muscle mass measured by computed tomography, or recent history of unintentional weight loss. We hypothesized that distinct metabolomic signals would be associated with worse HGS and SCP performance, which would be distinguishable from those associated with weight loss or lower muscle mass.

## 2 Methods

### 2.1 Design and Participants

This cross‐sectional study was conducted at the Veterans Affairs Puget Sound Health Care System (VAPSHCS) in Seattle, WA, USA. The protocol was approved by the VAPSHCS Institutional Review Board committee #1 and the Research and Development Committee and was conducted in compliance with the Declarations of Helsinki and its amendments and the International Conference on Harmonization Guideline for Good Clinical Practices.

Males with histologically, cytologically, or image‐based documented gastrointestinal or genitourinary cancer planning elective laparotomy were recruited from oncology or urology clinics at VAPSHCS. Participants were excluded for other conditions associated with cachexia (e.g., congestive heart failure, liver disease [aspartate aminotransferase or alanine aminotransferase equal or more than 3x normal levels], renal failure [creatinine equal or more than 2.5 mg/dL]), active infection, uncontrolled diabetes mellitus (HbA1c ≥ 9%), or intentional weight loss ≥ 5% within the prior 6 months.

### 2.2 Study Visit

Within 2 weeks prior to their scheduled surgery, participants reported to the VAPSHCS after a night of fasting; they were also asked to refrain from strenuous activity for 24 h prior to the study visit. A blood sample was obtained in EDTA‐containing vacutainers before 10 AM to measure plasma metabolites. Vacutainers were inverted 8–10 times, spun at room temperature for 10 min at 3500 rpm, and the plasma layer was extracted for storage at −80°C until analysis.

Objective physical function was measured by HGS (Jamar Hydraulic Dynamometer, J.A. Preston Corp., Clifton, NJ). Up to three trials were attempted for each hand, with the highest value per hand used to calculate mean HGS (kg) of both hands. For SCP, participants ascended a flight of standard hospital stairs (13 steps, 15.3 cm each) at the highest speed safely possible according to their capabilities [23]. Up to three trials were attempted, with the shortest time used to calculate power: Watts = (body mass [kg] × gravitational acceleration [9.81 m/s^2^] × vertical distance [1.99 m])/time (s).

### 2.3 Skeletal Muscle Collection

Rectus abdominis biopsies (0.5–1.0 g) were collected via transverse incision and sharp dissection during laparotomies. Specimens were quickly and grossly dissected to remove cauterized edges or vascular tissue, then immediately flash frozen using liquid nitrogen and stored at −80°C until analysis.

### 2.4 Computed Tomography Analysis

Clinically available spiral computed tomography scans involving L3 assessed prior to surgery were obtained from patients' electronic medical records. Cross‐sectional area (CSA; cm^2^) of skeletal muscle (psoas, paraspinals [quadratus lumborum, erector spinae], abdominals [lateral and oblique], and rectus abdominis) was quantified by research staff (L.A.) using image analysis sliceOmatic software (v5.0, TomoVision, Montreal, Quebec, Canada) with attenuation parameters −29 to 150 Hounsfield Units [24].

### 2.5 Metabolomics

Approximately 1.0 mg rectus abdominis and 25 μL plasma were sent to the Northwest Metabolomics Research Center at the University of Washington for performance of targeted metabolomics. In brief, targeted liquid chromatography mass spectrometry using a HILIC column to separate polar, aqueous metabolite analysis was performed. The assay targets over 350 metabolites from more than 60 metabolic pathways using a Sciex 6500 + platform and Aciex Analyst software for peak integration. All mass spectrometry experiments also included blank samples run under identical conditions to identify any signals arising from solvents/reagents/buffers that may also affect batch‐to‐batch variation. Plasma metabolite abundance was reported as absolute concentration while muscle metabolite abundance was normalized to protein loading and expressed as relative concentration. Patients were included in the present analysis if either plasma or muscle metabolome data were available. This data is available at the NIH Common Fund's National Metabolomics Data Repository (NMDR) website, the Metabolomics Workbench, https://www.metabolomicsworkbench.org where it has been assigned Study ID ST004161 (plasma data) and Study ID ST004166 (muscle data). The data can be accessed directly via Project DOI: https://doi.org/10.21228/M8QR9D (plasma data) or https://doi.org/10.21228/M86R9R (muscle data) [25].

### 2.6 Statistical Analysis

Data analysis was performed in the R statistical environment (R Core Team, 2024). Descriptive statistics of demographic variables, physical function, muscle mass, and weight loss were reported as median (interquartile range) or *N* (%). Metabolites with more than 25% missing values were excluded from analysis. Metabolite abundance was log_2_‐transformed to improve normality, and the remaining missing values were imputed using the K‐Nearest‐Neighbours algorithm (K = 5) with the R package ‘impute’ (version 1.70.0). To evaluate global metabolic variation associated with weight loss, low muscle area, or poor functional performance, multivariate analyses including partial least squares discriminant analysis (PLS‐DA, with the R package ‘mixOmics’, version 6.30.0) were performed.

Mann–Whitney U‐test was used to compare metabolite abundance between weight‐losing (6‐month history: ≥5% weight loss [1]) versus weight‐stable patients; analysed as a binary outcome. Spearman's correlation coefficients were computed to assess associations between L3‐CSA, HGS, or SCP and metabolite abundance; analysed as continuous outcomes. Compound identifiers and pathway membership information were provided by the Northwest Metabolomics Research Center. Pathway analysis was conducted using globaltest, a functional class scoring method that tests for differential pathways, with the R package ‘globaltest’ (version 5.50.0). A Benjamini‐Hochberg adjusted *p*‐value < 0.1 was used to control for false discovery rate.

## 3 Results

### 3.1 Participants

As depicted in Tables 1 and 2, the cohort (*N* = 72) was comprised of 70.8% white, non‐Hispanic males (*N* = 51) and 31.9% (*N* = 23) displayed unintentional weight loss ≥ 5% over the previous 6 months. There was a slightly greater proportion of genitourinary than gastrointestinal cancer, with 76.4% diagnosed with stage I/II tumours. Most patients did not receive chemotherapy and/or radiotherapy, 84.7% and 90.3%, respectively, within 3 months prior to study enrollment. Plasma and/or muscle metabolomics were evaluated in *N* = 68 and *N* = 64 patients, respectively, of the total *N* = 72 patients included here. A STROBE diagram is provided as supplementary materials (Figure S1) for an overview of comparisons and sample sizes.

**TABLE 1:** Patient demographics.

Med (IQR) or *N* (%)	Whole cohort (*N* = 72)	Plasma cohort (*N* = 68)	Muscle cohort (*N* = 64)
Age (years)	65.0 (59.0, 70.0)	65.0 (58.3, 70.0)	65.5 (59.2, 70.0)
Ht (cm)	177.8 (172.7, 182.9)	177.8 (172.7, 183.8)	177.8 (172.7, 182.9)
Wt (kg)	88.3 (78.1, 106.7)	88.3 (78.1, 107.3)	86.8 (77.4, 104.3)
BMI (kg/m^2^)	29.1 (26.6, 32.8)	29.1 (26.6, 32.8)	29.0 (25.0, 32.5)
**Ethnicity, *N* (%)**			
White, Non‐Hispanic	51 (70.8)	49 (72.1)	47 (73.4)
White, Hispanic	4 (5.6)	3 (4.4)	1 (1.6)
Black/African American	8 (11.1)	8 (11.8)	7 (10.9)
Asian/Native Hawaiian/Pacific Islander	4 (5.6)	4 (5.9)	4 (6.3)
Native American	2 (2.8)	1 (1.5)	2 (3.1)
Mixed	2 (2.8)	2 (2.9)	2 (3.1)
Unknown/Declined	1 (1.4)	1 (1.5)	1 (1.6)
**Tumour, *N* (%)**			
Gastrointestinal	31 (43.1)	27 (39.7)	28 (43.8)
Genitourinary	41 (56.9)	41 (60.3)	36 (56.3)
**Stage, *N* (%)**			
I	21 (29.2)	18 (26.5)	21 (32.8)
II	34 (47.2)	34 (50.0)	28 (43.8)
III/IV	17 (23.7)	16 (23.5)	15 (23.4)
**Comorbidities, N (%)**			
Diabetes	20 (27.8)	18 (26.5)	19 (29.7)
Hypertension	39 (54.2)	36 (52.9)	37 (57.8)
Hyperlipidaemia	29 (40.3)	27 (39.7)	25 (39.1)

*Note:* Group medians and interquartile ranges for patients with cancer in the whole cohort and for the sub‐groups with plasma metabolomics or muscle metabolomics.

Abbreviations: BMI, body mass index; Ht, height; Wt, weight.

**TABLE 2:** Patient characteristics.

Med (IQR) or N (%)	Whole cohort (*N* = 72)	Plasma cohort (*N* = 68)	Muscle cohort (*N* = 64)
Prior 6‐month Wt Loss ≥ 5%, *N* (%)	23 (31.9%)	23 (33.8%)	20 (31.3%)
CT‐muscle mass: L3‐CSA (cm^2^)	159.8 (144.4, 183.2) *N* = 47	159.3 (141.8, 183.3) *N* = 45	159.8 (144.4, 183.2) *N* = 43
Handgrip strength (kg)	36.1 (27.9, 41.8) *N* = 58	36.4 (29.9, 42.6) *N* = 54	36.1 (26.8, 40.5) *N* = 52
Stair climb power (W)	407.3 (327.6, 538.3) *N* = 25	413.2 (335.1, 547.7) *N* = 24	413.2 (335.1, 547.7) *N* = 24
Clinical laboratory values			
Haemoglobin (g/dL)	13.7 (12.9, 14.8) *N* = 66	13.7 (12.9, 14.8) *N* = 62	13.8 (13.0, 14.9) *N* = 58
Haematocrit (%)	40.5 (38.8, 43.9) *N* = 66	40.4 (38.8, 43.9) *N* = 62	40.6 (39.0, 44.2) *N* = 58
Leukocytes (K/μL)	7.4 (6.0, 8.9) *N* = 66	7.2 (6.0, 8.9) *N* = 62	7.4 (6.1, 8.7) *N* = 58
Platelets (K/μL)	244.0 (191.0, 296.0) *N* = 67	244.0 (191.0, 296.0) *N* = 63	244.0 (191.0, 308.0) *N* = 59
Albumin (g/dL)	4.3 (3.9, 4.6) *N* = 44	4.4 (4.0, 4.6) *N* = 42	4.3 (4.0, 4.6) *N* = 41

*Note:* Group medians and interquartile ranges for patients with cancer in the whole cohort and for the sub‐groups with plasma metabolomics or muscle metabolomics.

Abbreviations: CT, computed tomography; kg, kilograms; L3‐CSA, third lumbar cross‐sectional area; W, watts.

### 3.2 Global Metabolomic Signatures Associated With Cancer Cachexia‐Related Outcomes

**FIGURE 1:**
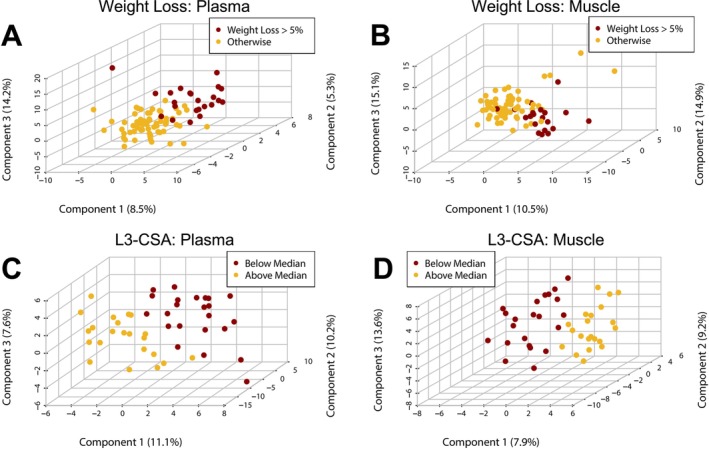
Partial least squares discriminant analysis of plasma (A, C) or skeletal muscle (B, D) metabolites associated with weight loss (A, B) or lower muscularity (C, D).

**FIGURE 2:**
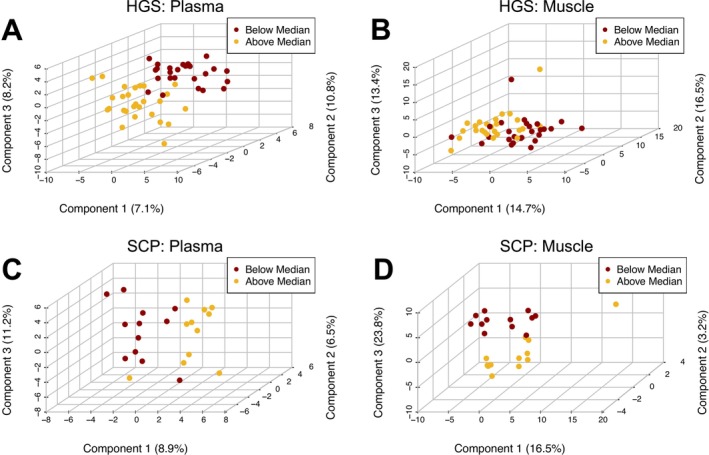
Partial least squares discriminant analysis of plasma (A, C) or skeletal muscle (B, D) metabolites associated with worse handgrip strength ‘HGS’ (A, B), or worse stair climb power ‘SCP’ (C‐D). A (below median: *N* = 27, equal to/above median: *N* = 27), B (below median: *N* = 26, equal to/above median: *N* = 26), C‐D (below median: *N* = 12, equal to/above median: *N* = 12).

After excluding metabolites with more than 25% missing values, 154 metabolites were evaluated in plasma and 138 were evaluated in skeletal muscle. The most abundant metabolites in plasma (top 10% ranked by median absolute concentration) were glutamine, urate, acetylcarnitine, histidine, carnitine, lysine, lactate, arginine, phenylalanine, leucine/D‐norleucine, glucose, creatinine, choline, citrulline, and glutamic acid. The most abundant metabolites in skeletal muscle (top 10% ranked by median relative concentration) were creatine, carnosine, glycerophosphocholine, acetylcarnitine, glucose‐6‐phosphate, glutamine, inosine monophosphate, carnitine, cadaverine, G1P/F1P/F6P, lactate, 4‐guanidinobutanoate, arginine, and reduced glutathione. Distinct clustering of subjects based on their global metabolomic profiles was observed in plasma and skeletal muscle with weight loss (Figure 1A,B), low muscularity (Figure 1C,D), poor HGS (Figure 2A,B), and poor SCP (Figure 2C,D). We observed similar clustering of global metabolomic profiles to low muscularity when assessing low skeletal muscle index. To avoid redundancy, these results have been provided as supplementary materials (Figure S2).

**FIGURE 3:**
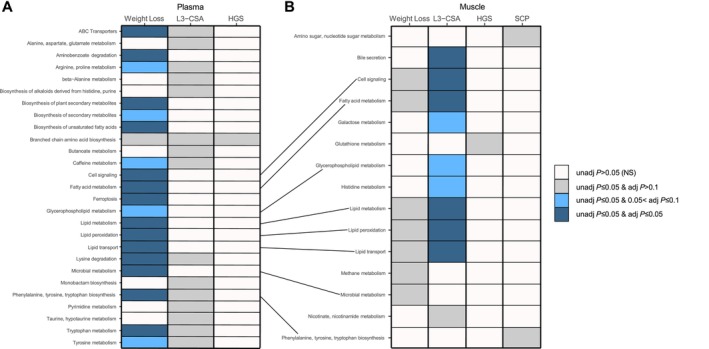
Overview of functional pathways altered in plasma (A) or skeletal muscle (B) with weight loss (A: weight loss, *N* = 23; weight stable, *N* = 45; B: weight loss, *N* = 20; weight stable, *N* = 44), lower muscularity (L3‐CSA; A: below median, *N* = 22; equal to/above median, *N* = 23; B: below median, *N* = 22; equal to/above median: *N* = 21), worse handgrip strength (HGS; A: below median: *N* = 27, equal to/above median: *N* = 27; B: below median: *N* = 26, equal to/above median: *N* = 26), or worse stair climb power (SCP; A‐B: below median: *N* = 12, equal to/above median: *N* = 12). AMP, adenosine monophosphate; CDP, cytidine diphosphate.

After excluding pathways with two or fewer probed metabolites, a total of 50 and 46 metabolic pathways were analysed in plasma and muscle, respectively. An overview summary of functional pathway alterations in plasma (Figure 3A) or skeletal muscle (Figure 3B) from weight loss, low muscularity, poor HGS, and/or poor SCP are represented by a summary heatmap. Further details on the metabolomic signatures of these measures are provided below.

### 3.3 Skeletal Muscle and Plasma Metabolomic Alterations Associated With Cancer‐Related Weight Loss

**FIGURE 4:**
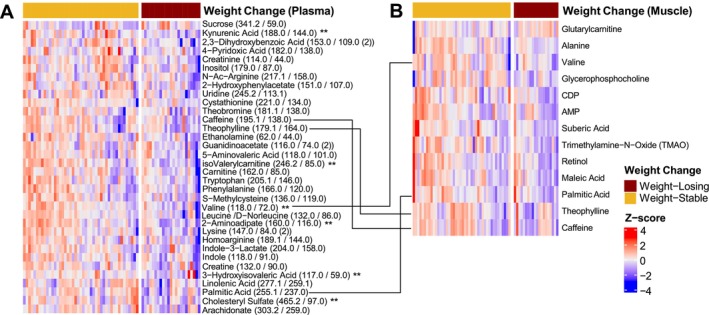
Heatmap of metabolites in plasma (A) or skeletal muscle (B) with differential abundance (unadjusted *p*‐value ≤ 0.05) between weight‐stable (A: *N* = 45, B: N = 44) and weight‐losing (A: N = 23, B: *N* = 20) patients. Adjusted *p*‐value ** < 0.1.

In patients with plasma metabolomics, *N* = 23 displayed > 5% weight loss over the prior 6 months [median (interquartile range): −7.6% (−12.1, −5.6)], while *N* = 45 displayed stable weight −0.7% (−2.1, +0.4). Patients with weight loss displayed significantly lower body mass index 27.4 (21.8, 31.7) kg/m^2^ than weight‐stable patients 29.8 (28.2, 33.7) kg/m^2^; *p* = 0.014. In univariate analysis, abundances of 33 metabolites in plasma were nominally lower (unadjusted *p* ≤ 0.05) while sucrose abundance was nominally higher (unadjusted *p* = 0.045) in weight‐losing than weight‐stable patients (Figure 4A). Isovalerylcarnitine, 2‐aminoadipate, kynurenic acid, 3‐hydroxyisovaleric acid, cholesteryl sulfate, and valine remained significant after adjustment for multiple testing (adjusted *p* < 0.1).

**FIGURE 5:**
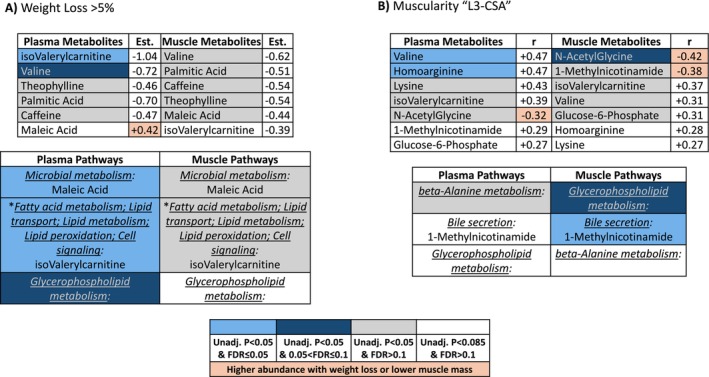
Metabolites and pathways listed represent those consistently altered (at minimum an unadjusted *p* < 0.05 in plasma or skeletal muscle, and *p* < 0.085 in the other compartment) in patients with weight loss (A) or lower lumbar muscle cross‐sectional area ‘L3‐CSA’ (B). Consistently altered metabolites are listed with their corresponding pathways, as applicable. *Multiple pathways listed, separated by semicolon. Est, estimate (Mann Whitney U‐test: positive = higher abundance in weight loss versus weight stable); r, correlation coefficient (Spearman; negative = higher abundance with lower muscle cross‐section); Unadj, unadjusted; FDR, false discovery rate.

In patients with muscle metabolomics, *N* = 20 displayed > 5% weight loss over the prior 6 months [median (interquartile range): −7.1% (−10.2, −5.5)], while *N* = 44 displayed stable weight −0.7% (−2.3, +0.6). Patients with weight loss displayed significantly lower body mass index 26.6 (20.9, 31.0) kg/m^2^ than weight‐stable patients 29.5 (27.7, 33.5) kg/m^2^; *p* = 0.014. In skeletal muscle, abundances of 12 metabolites were nominally lower (unadjusted *p* ≤ 0.05) while glutarylcarnitine abundance was nominally higher (unadjusted *p* = 0.024) in weight‐losing than weight‐stable patients (Figure 4B). None were significant after adjustment for multiple testing. Valine, isovalerylcarnitine, theophylline, palmitic acid, caffeine, and choline abundance was lower with weight loss in both plasma and skeletal muscle; maleic acid abundance was also lower in skeletal muscle but higher with weight loss in plasma (Figure 5).

Pathway analysis identified 20 metabolic processes that were nominally altered in plasma (unadjusted *p* ≤ 0.05) and most remained significant after adjustment for multiple testing (adjusted *p* ≤ 0.1; Figure 3A). Seven pathways were nominally altered in skeletal muscle (unadjusted *p* ≤ 0.05), but none were significant after adjustment for multiple testing (Figure 3B). Microbial metabolism, fatty acid metabolism, lipid transport, lipid metabolism, lipid peroxidation, cell signalling, glycerophospholipid metabolism, biosynthesis of plant secondary metabolites, and biosynthesis of alkaloids derived from histidine/purine were altered in both plasma and skeletal muscle. Univariate analysis of metabolite abundance and complete globaltest pathway results for weight‐losing versus weight‐stable are available in Table S1.

### 3.4 Skeletal Muscle and Plasma Metabolomic Alterations Associated With Low Muscle Mass in Cancer

**FIGURE 6:**
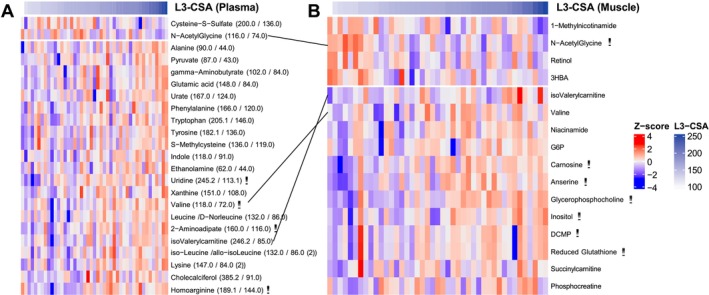
Heatmap of metabolites in plasma (A) or skeletal muscle (B) that are correlated (unadjusted *p*‐value ≤ 0.05) with lumbar cross‐sectional area (A: N = 45, B: *N* = 43). Adjusted *p*‐value ** < 0.1.

In univariate association, abundances of 21 plasma metabolites were nominally positively correlated (unadjusted *p* ≤ 0.05) with L3‐CSA; 2‐aminoadipate, homoarginine, uridine, and valine remained significant after adjustment for multiple testing (*r* = 0.47–0.50, adjusted *p* < 0.1; Figure 6A). Abundance of Cysteine‐S‐Sulfate and N‐AcetylGlycine was nominally negatively correlated (unadjusted *p* ≤ 0.05) with L3‐CSA (Figure 6A). In skeletal muscle, abundances of 12 metabolites were nominally positively correlated (unadjusted *p* ≤ 0.05) with L3‐CSA; glycerophosphocholine, carnosine, anserine, inositol, reduced glutathione, and deoxycytidine monophosphate remained significant after adjustment for multiple testing (*r* = 0.42–0.68, adjusted *p* < 0.1; Figure 6B). In skeletal muscle, abundances of five metabolites were nominally negatively correlated (unadjusted *p* ≤ 0.05) with L3‐CSA; N‐acetylglycine remained significant after adjustment for multiple testing (*r* = −0.42, adjusted *p* = 0.095; Figure 6B). Isovalerylcarnitine and valine were each positively correlated while N‐acetylglycine was negatively correlated with L3‐CSA in both plasma and skeletal muscle (Figure 5). We observed similar metabolic signals when using skeletal muscle index (Tables S4 and S5).

Fifteen pathways were nominally altered in plasma (unadjusted *p* ≤ 0.05), but none were significant after adjustment for multiple comparisons (Figure 3A). Ten pathways were nominally altered in skeletal muscle (unadjusted *p* ≤ 0.05) and nine remained significant after adjustment for multiple comparisons (adjusted *p* < 0.1; Figure 3B). Bile secretion was nominally altered in both plasma and muscle and remained significant after adjustment for multiple testing in muscle (adjusted *p* = 0.022). Univariate association and complete globaltest pathway results for L3‐CSA are available in Table S2.

### 3.5 Skeletal Muscle and Plasma Metabolomic Alterations With Low Handgrip Strength in Cancer

In univariate association, plasma abundances of indole‐3‐propionate, ergocalciferol, theophylline, threonine, pipecolate, deoxycarnitine, caffeine, glycine, and 4‐guaninidnobutanoate were nominally positively correlated with HGS (*r* = 0.27–0.45, unadjusted *p* ≤ 0.05). Only indole‐3‐propionate remained significant after adjustment for multiple comparisons (*r* = 0.45, adjusted *p* = 0.09). In skeletal muscle, oxidized glutathione was nominally positively correlated (*r* = 0.29, unadjusted *p* = 0.034), while gamma‐aminobutyrate, succinate, and 5′‐methylthioadenosine were nominally negatively correlated (*r* = ‐0.30, unadjusted *p* ≤ 0.05) with HGS; none remained significant after correction for multiple comparisons.

Branched chain amino acid biosynthesis was nominally altered in plasma (unadjusted *p* = 0.018; Figure 3A) and glutathione metabolism was nominally altered in skeletal muscle (unadjusted *p* = 0.045; Figure 3B) but neither reached significance after correction for multiple comparisons. Univariate associations and complete globaltest results for HGS are available in Table S3.

### 3.6 Skeletal Muscle and Plasma Metabolomic Alterations With Low Stair Climb Power in Cancer

In univariate association, plasma abundance of cadaverine was nominally positively correlated (*r* = 0.48, unadjusted *p* = 0.019), while fructose (*r* = −0.44, unadjusted *p* = 0.03) and succinate (*r* = −0.41, unadjusted *p* = 0.048) were nominally negatively correlated with SCP. In skeletal muscle, abundances of guanidinoacetate, glycine, carnosine, anserine, phenylalanine, leucine/D‐norleucine, iso‐leucine/allo‐isoleucine, histamine, glucosamine‐6‐phosphate, UDP‐GlcNAc, and methionine were nominally positively correlated (*r* = 0.41–0.57, unadjusted *p* ≤ 0.05) with SCP. No correlations in plasma or skeletal muscle reached significance after adjustment for multiple comparisons.

No significantly altered pathways linked to SCP were detected in plasma. Amino sugar and nucleotide sugar metabolism (unadjusted *p* = 0.018) and phenylalanine, tyrosine, and tryptophan biosynthesis (unadjusted *p* = 0.045) pathways were nominally altered in muscle (Figure 3B), but these did not remain significant after adjustment for multiple comparisons. Univariate associations and complete globaltest results for SCP are available in Table S3.

## 4 Discussion

This is the first study to concurrently characterize metabolic perturbations in plasma and skeletal muscle associated with cachexia measures that go beyond body weight changes to include muscle mass and function. Most plasma pathways altered with weight loss, which were related to numerous cellular processes, remained significant after adjustment for multiple hypothesis testing. Most skeletal muscle pathways altered with low muscularity were primarily related to metabolism of fatty acids/lipids and remained significant after adjustment. Nominally, significant skeletal muscle metabolic signatures were detected with worse HGS or SCP that were distinguishable from the skeletal muscle profiles associated with weight loss or lower CSA. In plasma, branched chain amino acid biosynthesis was nominally altered with poorer HGS, which was also nominally altered in plasma with weight loss and lower CSA but attributed to different metabolite alterations. Plasma indole‐3‐propionate was positively correlated with HGS, and this association remained significant after adjustment for multiple comparisons.

Alterations in the circulating metabolome with cancer‐related weight loss have been primarily examined in mixed tumour cohorts [15, 19, 20]. Our findings are consistent with these studies which reported lower abundance of lysine, valine [15, 20] phenylalanine, tryptophan, indole‐3‐lactate [15], carnitine [15, 19], and/or leucine [19, 20] in weight‐losing patients. One of these studies reported alterations in the arginine/proline metabolism pathway and the phenylalanine, tyrosine, and tryptophan biosynthesis pathway [20], which we also observed here. Another of these studies reported altered lysine degradation, branched chain amino acid biosynthesis, phenylalanine metabolism, and arginine/proline metabolism [19]. Despite the fact that weight loss‐associated metabolites were pooled from serum and urine to identify pathway perturbations in that study [19], we observed consistent findings in our plasma analyses here. These observations imply that poor circulating availability of essential amino acids such as lysine, valine, leucine, phenylalanine, and tryptophan is associated with cancer‐related weight loss.

In contrast, lower CSA in the current study was primarily associated with altered skeletal muscle fatty acid, lipid, histidine, and glycerophospholipid metabolism. Upregulation of skeletal muscle fatty acid oxidation has been observed in murine models of cancer cachexia, largely in association with elevated ketone bodies [26]. In humans, elevated serum 3‐hydroxybutyrate, a ketone body and byproduct of fatty acid metabolism, was identified as a predictor of weight loss during radiotherapy or chemoradiotherapy [27]. We observed elevated skeletal muscle 3‐hydroxybutyrate levels with lower CSA, indicating elevated fatty acid oxidation, but observed no effect on plasma abundance and no effect of weight loss on plasma or muscle abundance of 3‐hydroxybutyrate.

Glycerophosphocholine is a precursor for ethanolamine and choline which promote synthesis of multiple phospholipids that are critical for the integrity and function of sarcolemmal and mitochondrial membranes. Perturbations in skeletal muscle glycerophospholipid metabolism are reportedly associated with age, insulin resistance, and impaired glucose metabolism in older adults [28] and with impaired glucose metabolism and cross‐bridge cycling in mice [29]. Skeletal muscle glycerophosphocholine and plasma ethanolamine were positively correlated with CSA and were lower with weight loss in the current study. This may indicate that cachectic patients are unable to meet the phospholipid requirements for maintaining structural health, glucose/insulin dynamics, or energy demand in skeletal muscle. Lower skeletal muscle and/or plasma phospholipid expression has been reported in other weight‐losing cancer patients [15, 16, 30].

We also found altered skeletal muscle histidine metabolism with low CSA, which was related to lower carnosine and anserine abundance. Altered histidine metabolism was previously observed in patients with pancreatic/periampullary cancer and low skeletal muscle index (CSA divided by height), though that was reported in plasma [21]. However, lower carnosine and anserine abundance in rectus abdominis was reported in patients with upper gastrointestinal cancer and low skeletal muscle index [31], suggesting that these metabolites are directly related to muscularity. Carnosine and anserine are both beta‐alanine containing dipeptides, and we observed nominally perturbed beta‐alanine metabolism in plasma with lower CSA.

These findings suggest that beta‐alanine supplementation should be investigated for a potential role in mitigation of cancer‐related muscle wasting. Amino acid supplementation in cancer cachexia clinical trials is often administered with other agents or interventions, which impedes determination of the contribution from amino acids on tumour activity [32, 33]. However, clinical trials administering protein/amino acid diets indicate that these interventions are beneficial in patients with cancer but should be monitored with extra caution in cancer patients with weight loss due to the largely unknown risk of tumour progression in that setting [32, 33].

Other potential supplementation strategies targeting skeletal muscle include 1‐Methylnicotinamide or glutathione. While these metabolites are mapped to the bile secretion pathway, they are involved in a number of processes in skeletal muscle. For example, during situations like fasting or endurance exercise when skeletal muscle energy stores are low, 1‐Methylnicotinamide is produced from nicotinamide metabolism and is released to stimulate lipolysis in white adipose tissue and gluconeogenesis in the liver [34]. Our finding of greater 1‐methylnicotinamide abundance with lower CSA is consistent with a muscular response to metabolic stress.

Reduced glutathione is a major antioxidant in skeletal muscle which maintains redox balance by scavenging reactive oxygen species and promotes contractility by detoxifying oxidative stress during exercise when ATP demand is high [35]. Therefore, we cannot rule out that the impact of these two metabolites is independent of one another due to their skeletal muscle‐specific actions. The positive correlation between the abundance of reduced glutathione and CSA observed here, and the complementary roles of 1‐methylnicotinamide and glutathione for stimulating energy availability and mitigating oxidative stress, warrant investigation of these targets for improving muscular health and performance in cancer cachexia.

The complementary roles of 1‐methylnicotinamide and glutathione for stimulating energy availability and mitigating oxidative stress during metabolic dysregulation make these metabolites intriguing targets for improving muscular health and performance in cancer cachexia. Supplementation with compounds that increase NAD+, which is metabolized to nicotinamide, improved physical function in middle‐aged healthy adults and in patients with amyotrophic lateral sclerosis [36] but the effect on cancer cachexia has not been examined. Glutathione improved neurotoxicity during platinum‐based chemotherapy for gastric, colorectal, or ovarian cancer [37], but its impact on physical function or muscle mass remains largely untested.

Furthermore, the positive correlation between HGS and skeletal muscle abundance of oxidized glutathione observed here supports the premise that glutathione availability may be important for functional performance. Lower total glutathione levels in vastus lateralis along with worse gait speed, chair‐stand, and 6‐min walk performance were reported in older compared to younger adults; oxidized glutathione levels were not measured [38]. Leakage of oxidized glutathione into the blood may promote an antioxidant/redox imbalance within the muscle. This is consistent with reported elevations of oxidized glutathione levels in red blood cells along with slower gait speed in older adults with sarcopenia (low HGS plus low muscle mass) versus those without sarcopenia [39]. We did not measure red blood cell oxidized glutathione, which is likely why we did not detect a correlation between its abundance and HGS in plasma.

Instead, we observed nominally altered branched‐chain amino acid biosynthesis in plasma with HGS. This is likely due to the lower abundance of threonine, which is a central precursor to this pathway. Lower plasma threonine was reported in older adults with sarcopenia (low gait speed and/or leg strength plus low muscle mass) [40] or possible sarcopenia (low HGS only) [41] versus those without sarcopenia. The possible sarcopenia group also displayed worse chair stand, 6‐min walk, 30‐s arm curl, 2‐min step, and timed up‐and‐go performance, but appendicular skeletal muscle index was similar to the non‐sarcopenic group [41]. These data highlight the disconnect between muscle mass and muscle function and suggest that poor functional performance may be reflected in the circulation by lower threonine abundance.

Indole‐3‐propionate, produced during tryptophan metabolism by gut microbes, was also significantly correlated with HGS. Its positive association with insulin sensitivity and inverse association with inflammatory markers in adults with obesity or Type II diabetes along with its purported benefit in mitochondria, liver, and intestines was recently reviewed [42]. Treatment with indole‐3‐propionate activated myogenic regulatory factors in myoblasts and reduced inflammation in myotubes, indicating potential mechanisms of benefit in skeletal muscle as well [43].

We observed a nominally positive correlation between SCP and plasma cadaverine, a byproduct of microbial lysine metabolism which is typically produced in decaying tissue. Lower plasma abundance with lower SCP may suggest that circulating lysine availability is important for muscular power production. In contrast, fructose and succinate were nominally negatively correlated with lower SCP. Elevated circulating fructose and succinate with lower SCP may be related to the blunted skeletal muscle amino sugar/nucleotide sugar metabolism and phenylalanine/tryptophan metabolism, respectively, in skeletal muscle observed here. These observations represent the first report of circulating and skeletal muscle metabolomic perturbations with worse SCP performance.

Our study has several limitations including its moderate sample size which reduced power and impeded our ability to determine the independent effect of diagnostic parameters such as tumour type or stage. We used an adjusted *p*‐value < 0.1 to designate significance but also reported results with nominally significant *p*‐values (< 0.05) as exploratory findings. Therefore, our current results, particularly those related to worse HGS and SCP, will require validation by larger studies with adequate statistical power to detect significance after adjustment for multiple comparisons. A more comprehensive metabolic profiling platform can identify additional metabolites associated with functional impairment in cancer. It is important to note that perturbations detected in cross‐sectional analyses may not reflect those detected longitudinally, and alterations associated with cachexia may differ when induced by tumour burden or from cancer treatment. We also cannot rule out influence from concomitant/recent non‐prescribed supplements and/or alternative therapies that were not captured here. For example, although participants were asked to fast overnight, caffeine or its metabolites, including theophylline, were detectable here. This may be due to caffeine ingestion acutely before the study visit and/or to slow metabolism, but no patients were actively taking theophylline as a prescription medication.

The inclusion of only gastrointestinal and genitourinary tumours may limit the generalizability of our findings; although our observations were consistent with other studies involving various cancers, indicating that tumour type may not be a major determinant of cachexia‐related metabolic perturbations. Moreover, we cannot rule out that our findings reflect the cancer diagnosis and not of weight loss and/or poor function or muscle mass, or that patients did not display these factors prior to cancer diagnosis. However, these limitations are common to most clinical trials in this setting as such retrospective details are nearly impossible to obtain without extensive longitudinal observation or using a prospective randomized design. We also cannot rule out that these findings are specific to males since females were not eligible for inclusion in this study. As Veterans are overwhelmingly male, the small number of females that may have been recruited would likely not be sufficient to test whether sex was a confounding factor.

In summary, this is the first study to examine metabolomic alterations related to physical function in both plasma and skeletal muscle in cancer patients. The metabolomic signatures detected here were different between weight loss, low muscle mass, or worse physical function, which supports clinical observations indicating that different pathways govern these cancer‐related phenotypes. Pathways of interest for HGS include branched chain amino acid metabolism in plasma and glutathione metabolism in skeletal muscle. Pathways of interest for SCP include metabolism of amino sugars/nucleotide sugars, phenylalanine, histidine, glycine, and threonine in skeletal muscle. Weight loss was associated with altered plasma metabolism of fatty acid/lipids and numerous amino acids/derivatives, while low muscle was associated with altered fatty acid/lipid metabolism in skeletal muscle.

Future studies are needed to further characterize metabolic profiles related to various categories of functional performance such as muscular strength, power, fatigue, aerobic capacity and endurance, physical activity level, and functional daily activities. Furthermore, metabolomic perturbations associated with longitudinal changes in cachexia‐related outcomes remain to be determined. Better characterization of these alterations may identify biomarkers and pathways that can be targeted to improve functional impairments in cancer cachexia.

## Funding

The authors have nothing to report.

## Conflicts of Interest

The authors declare no conflicts of interest.

## Supporting information


**Figure S1:** STROBE diagram displaying the summary of samples sizes for comparison of study outcomes.

**Figure S2:** Partial least squares discriminant analysis of plasma **(A)** or skeletal muscle **(B)** metabolites associated with lower skeletal muscle index (SMI): L3 muscle cross‐sectional area/height (cm^2^).

**Table S1:** Metabolomic Alterations in Muscle and Plasma with Cancer‐Related Weight Loss.
**Table S2:** Metabolomic Alterations in Muscle and Plasma Associated with Low Muscularity in Cancer.
**Table S3:** Metabolomic Alterations in Muscle and Plasma Associated with Worse Physical Function in Cancer.

**Table S4:** Nominal Spearman's correlations between plasma metabolite abundances and skeletal muscle index.
**Table S5:** Nominal Spearman's correlations between muscle metabolite abundances and skeletal muscle index.
