# Supplementary figures and images for: Distinct Metabolomic Alterations Are Associated With Physical Function, Weight Loss, and Muscle Mass in Men With Cancer
Source: J Cachexia Sarcopenia Muscle. 2026 Jan 18;17(1):e70183. doi: 10.1002/jcsm.70183 (PMC12813416; doi:10.1002/jcsm.70183)

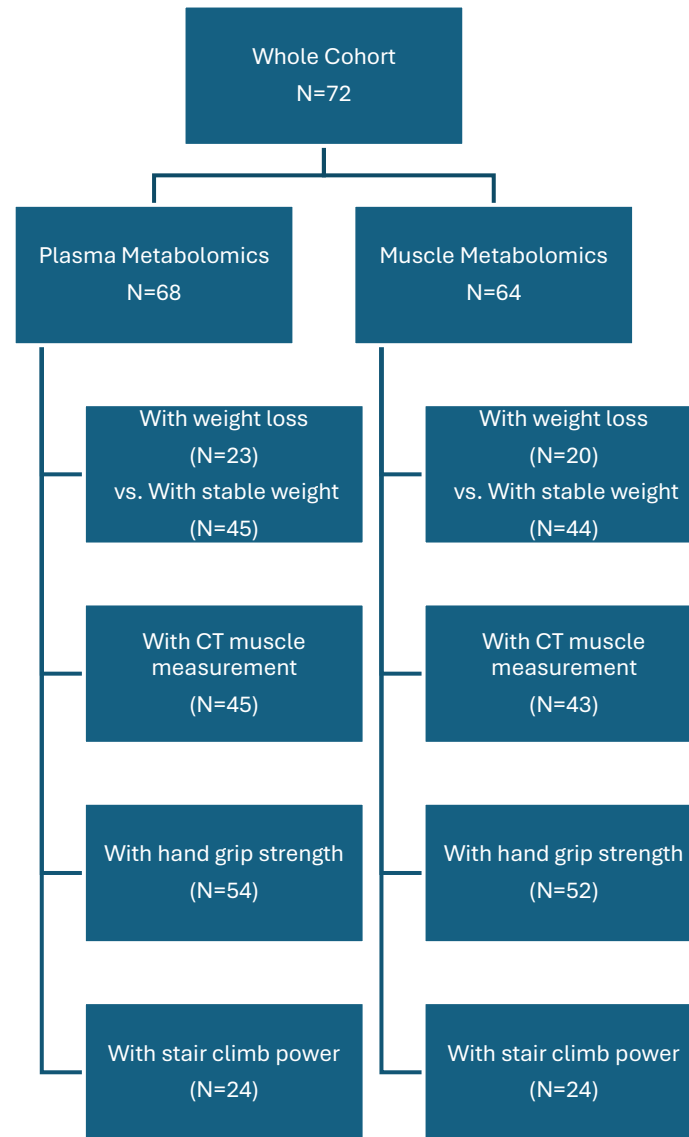

Supplement: Supplementary file 1 — Figure S1: STROBE diagram displaying the summary of samples sizes for comparison of study outcomes. [file JCSM-17-e70183-s002.pdf]

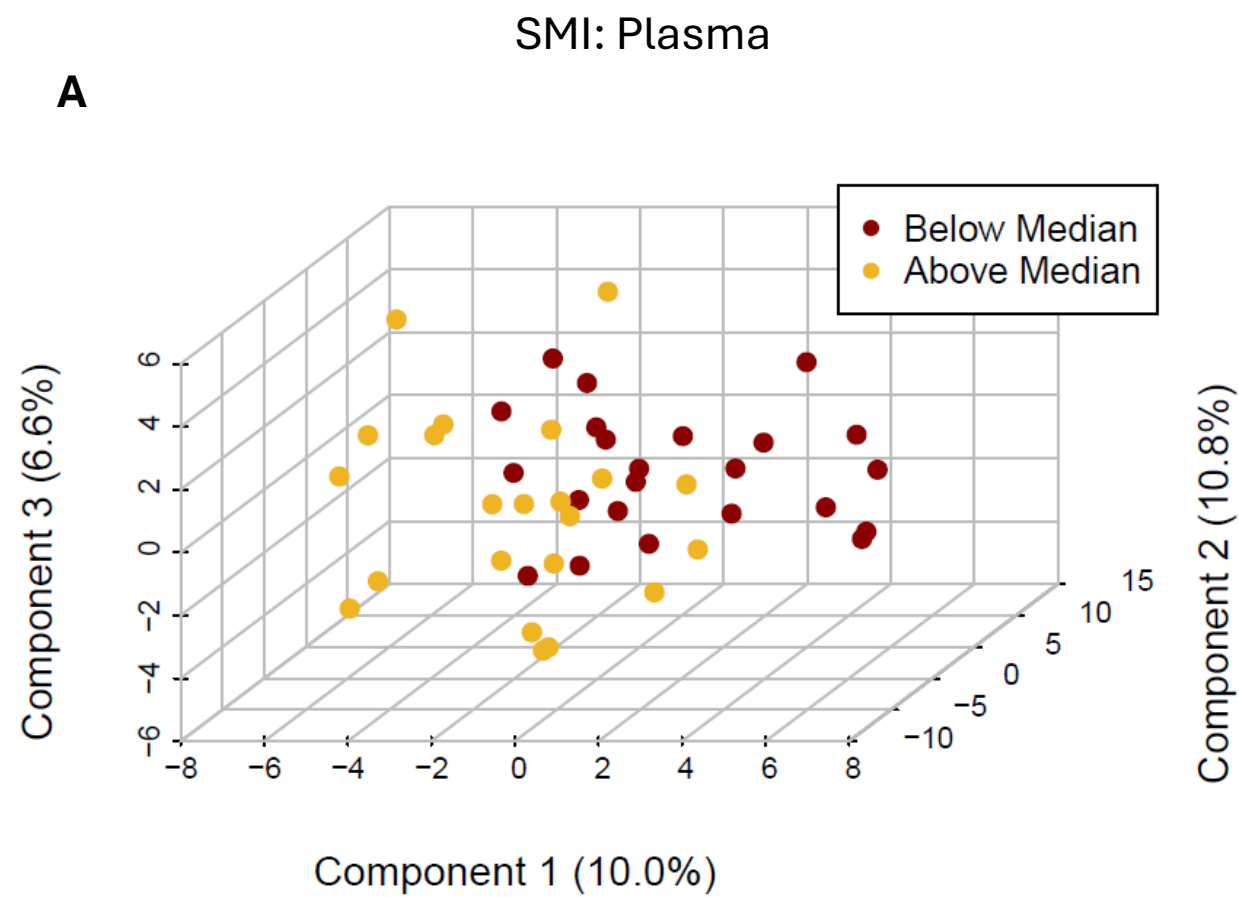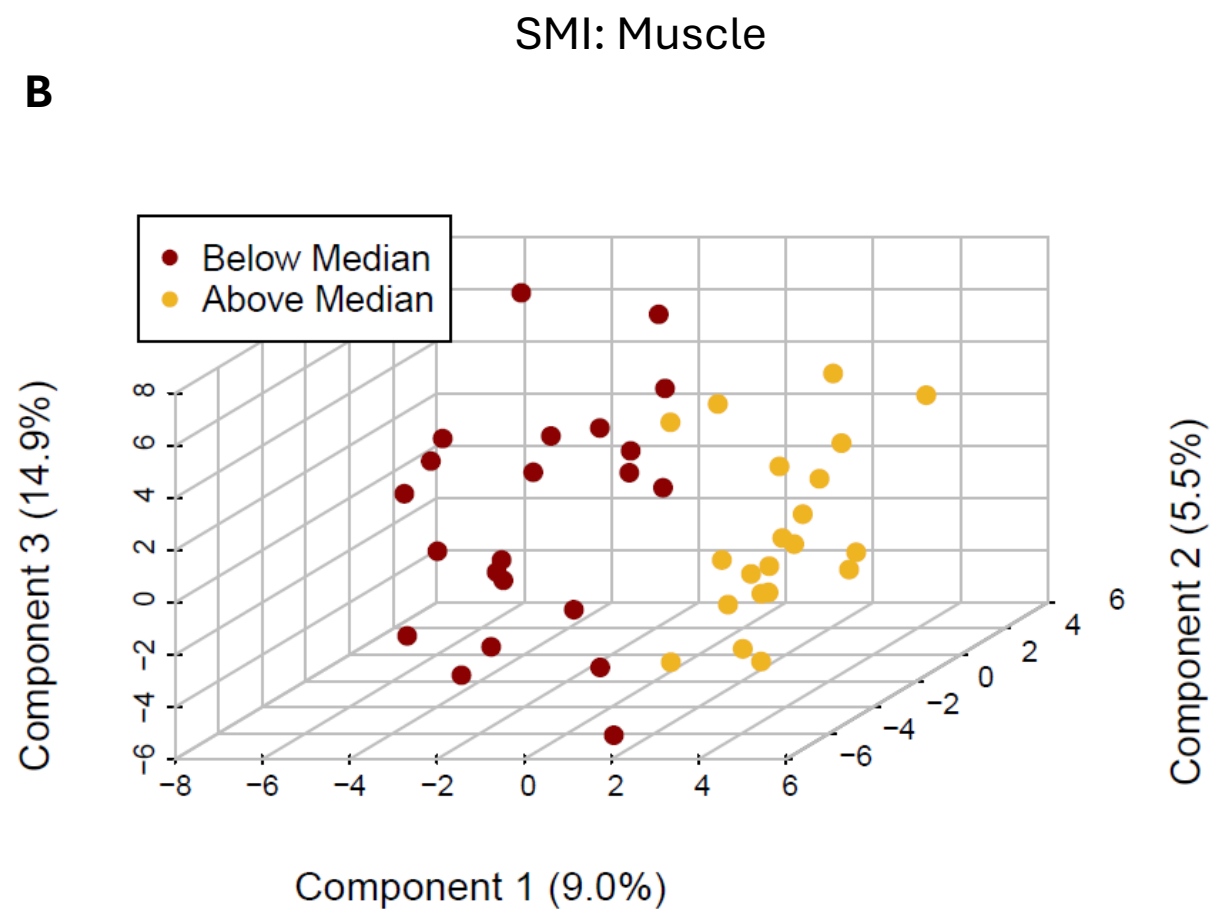

Supplement: Supplementary file 2 — Figure S2: Partial least squares discriminant analysis of plasma (A) or skeletal muscle (B) metabolites associated with lower skeletal muscle index (SMI): L3 muscle cross‐sectional area/height (cm2). [file JCSM-17-e70183-s003.pdf]
