# Supplementary material for: Distinct Metabolomic Alterations Are Associated With Physical Function, Weight Loss, and Muscle Mass in Men With Cancer
Source: J Cachexia Sarcopenia Muscle. 2026 Jan 18;17(1):e70183. doi: 10.1002/jcsm.70183 (PMC12813416; doi:10.1002/jcsm.70183)
Supplement: Supplementary file 3 — Table S1: Metabolomic Alterations in Muscle and Plasma with Cancer‐Related Weight Loss. Table S2: Metabolomic Alterations in Muscle and Plasma Associated with Low Muscularity in Cancer. Table S3: Metabolomic Alterations in Muscle and Plasma Associated with Worse Physical Function in Cancer. [file JCSM-17-e70183-s004.pdf]

Supplemental Table 1. Metabolomic Alterations in Muscle and Plasma with Cancer-Related Weight Loss

U-test	Log_2 (FC)	P-value	Adj. P-value	globaltest	# Det.	P-value	Adj. P-value
**Plasma Metabolites**				**Plasma Pathways**			
isoValerylcarnitine (246.2/85.0)	-1.04	**<0.001**	**0.004**	*Microbial metabolism:* 2,3-Dihydroxybenzoic Acid, 2-Hydroxyphenylacetate, 4-Pyridoxic Acid, 5-Aminovaleric Acid, Maleic Acid, Xanthine	12	**<0.001**	**0.010**
2-Aminoadipate (160.0/116.0)	-0.90	**<0.001**	**0.024**	*Aminobenzoate degradation:* 2,3-Dihydroxybenzoic Acid, 2-Hydroxyphenylacetate	3	**<0.001**	**0.010**
Kynurenic Acid (188.0/144.0)	-0.84	**<0.001**	**0.024**	*Tryptophan metabolism:* Kynurenic Acid, Tryptophan, Indole, Indole-3-Lactate	6	**0.001**	**0.013**
3-Hydroxyisovaleric Acid (117.0/59.0)	-0.65	**0.001**	**0.042**	*Cell signaling:* isoValerylcarnitine	4	**0.001**	**0.013**
Cholesteryl Sulfate (465.2/97.0)	-0.85	**0.002**	**0.054**	*Lysine degradation:* 2-Aminoadipate, Lysine, Carnitine, 5-Aminovaleric Acid, Homoarginine	10	**0.003**	**0.021**
Valine (118.0/72.0)	-0.72	**0.002**	**0.054**	*Fatty acid metabolism:* isoValerylcarnitine	7	**0.004**	**0.021**
Ethanolamine (62.0/44.0)	-0.78	**0.005**	0.108	*Lipid metabolism:* isoValerylcarnitine	7	**0.004**	**0.021**
Lysine (147.0/84.0 (2))	-0.73	**0.006**	0.108	*Lipid peroxidation:* isoValerylcarnitine	7	**0.004**	**0.021**
Leucine/D-Norleucine (132.0/86.0)	-0.63	**0.007**	0.118	*Lipid transport:* isoValerylcarnitine	7	**0.004**	**0.021**
Indole-3-Lactate (204.0/158.0)	-0.71	**0.008**	0.125	*Ferroptosis:* Arachidonate	4	**0.004**	**0.021**
2,3-Dihydroxybenzoic Acid (153.0/109.0 (2))	-0.59	**0.012**	0.143	*ABC Transporters:* Uridine, S-Methylcysteine, Carnitine, Sucrose, Choline	9	**0.006**	**0.026**
Tryptophan (205.1/146.0)	-0.64	**0.012**	0.143	*Phenylalanine, tyrosine, tryptophan biosynthesis:* Tryptophan, Indole, Phenylalanine, Indole-3-Lactate	4	**0.009**	**0.036**
Creatinine (114.0/44.0)	-0.64	**0.012**	0.143	*Biosynthesis of unsaturated fatty acids:* Arachidonate, Linolenic Acid	3	**0.010**	**0.037**
2-Hydroxyphenylacetate (151.0/107.0)	-0.70	**0.016**	0.158	*Biosynthesis of plant secondary metabolites:* Theophylline, Linolenic Acid, Theobromine, Caffeine, Xanthine	11	**0.010**	**0.037**
Arachidonate (303.2/259.0)	-0.76	**0.018**	0.158	*Biosynthesis of secondary metabolites:* 2,3-Dihydroxybenzoic Acid, Linolenic Acid, Sucrose, Cystathionine, Xanthine	11	**0.017**	**0.058**
Indole (118.0/91.0)	-0.58	**0.020**	0.158	*Caffeine metabolism:* Theophylline, Theobromine, Caffeine, Xanthine	4	**0.021**	**0.064**
Phenylalanine (166.0/120.0)	-0.56	**0.023**	0.158	*Tyrosine metabolism:* Maleic Acid, Tyrosine	4	**0.022**	**0.064**

Uridine (245.2/113.1)	-0.62	**0.023**	0.158	*Glycerophospholipid metabolism*:<br>Ethanolamine, Choline	4	**0.024**	**0.067**
4-Pyridoxic Acid (182.0/138.0)	-0.43	**0.024**	0.158	*Arginine, proline metabolism*:<br>Creatinine, Guanidinoacetate, Creatine, 5-Aminovaleric Acid, Homoarginine	13	**0.026**	**0.069**
Theophylline (179.1/164.0)	-0.46	**0.024**	0.158	*Branched chain amino acid biosynthesis*:<br>3-Hydroxyisovaleric Acid, Valine, Leucine/D-Norleucine, iso-Leucine/allo-isoLeucine	4	**0.042**	0.104
Homoarginine (189.1/144.0)	-0.68	**0.025**	0.158	*Phenylalanine metabolism*:<br>Phenylalanine	6	0.054	0.128
Linolenic Acid (277.1/259.1)	-0.62	**0.026**	0.158	*Biosynthesis of alkaloids derived from histidine, purine*:<br>Theophylline, Theobromine, Caffeine, Xanthine	6	0.069	0.150
Palmitic Acid (255.1/237.0)	-0.70	**0.026**	0.158	*Glycine, serine, threonine metabolism*:<br>Tryptophan, Cystathionine, Guanidinoacetate, Creatine, Choline	11	0.069	0.150
Cystathionine (221.0/134.0)	-0.41	**0.028**	0.158	*Butanoate metabolism*:<br>2,3-Dihydroxybenzoic Acid, Maleic Acid	4	0.095	0.197
Guanidinoacetate (116.0/74.0 (2))	-0.63	**0.028**	0.158				
Inositol (179.0/87.0)	-0.63	**0.028**	0.158				
N-Ac-Arginine (217.1/158.0)	-0.63	**0.028**	0.158				
Creatine (132.0/90.0)	-0.66	**0.031**	0.163				
S-Methylcysteine (136.0/119.0)	-0.66	**0.031**	0.163				
Theobromine (181.1/138.0)	-0.50	**0.034**	0.174				
Caffeine (195.1/138.0)	-0.47	**0.035**	0.174				
Carnitine (162.0/85.0)	-0.47	**0.040**	0.192				
5-Aminovaleric Acid (118.0/101.0)	-0.56	**0.044**	0.205				
Sucrose (341.2/59.0)	+0.40	**0.045**	0.205				
iso-Leucine/allo-isoLeucine (132.0/86.0 (2))	-0.60	0.051	0.220				
Myristic Acid (227.2/209.1)	-0.48	0.051	0.220				
Maleic Acid (115.0/71.0 (2))	+0.42	0.053	0.220				
Tyrosine (182.1/136.0)	-0.48	0.060	0.242				
5-Methyluridine (257.1/42.0)	-0.49	0.062	0.243				
1-Methyladenosine (282.2/150.1)	-0.45	0.067	0.259				
Choline (104.0/60.0)	-0.40	0.073	0.276				
Xanthine (151.0/108.0)	-0.31	0.092	0.331				

Skeletal Muscle Metabolites				Skeletal Muscle Pathways			
Glycerophosphocholine	-0.66	**0.002**	0.184	*Microbial metabolism:*<br>TMAO, Maleic Acid, Urate	6	**0.012**	0.138
Trimethylamine-N-Oxide "TMAO"	-0.81	**0.003**	0.184	*Fatty acid metabolism:*<br>Suberic Acid, Glutarylcarnitine, Retinol, isoValerylcarnitine, 2-Hydroxyisobutyrate/2-Hydroxybutyrate	5	**0.017**	0.138
Cytidine Diphosphate "CDP"	-0.73	**0.006**	0.270	*Lipid metabolism:*<br>Suberic Acid, Glutarylcarnitine, Retinol, isoValerylcarnitine, 2-Hydroxyisobutyrate/2-Hydroxybutyrate	5	**0.017**	0.138
Suberic Acid	-0.53	**0.012**	0.332	*Lipid peroxidation:*<br>Suberic Acid, Glutarylcarnitine, Retinol, isoValerylcarnitine, 2-Hydroxyisobutyrate/2-Hydroxybutyrate	5	**0.017**	0.138
Alanine	-0.41	**0.012**	0.332	*Lipid transport:*<br>Suberic Acid, Glutarylcarnitine, Retinol, isoValerylcarnitine	5	**0.017**	0.138
Valine	-0.62	**0.023**	0.417	*Cell signaling:*<br>Glutarylcarnitine, Retinol, isoValerylcarnitine	3	**0.018**	0.138
Palmitic Acid	-0.51	**0.023**	0.417	*Methane metabolism:*<br>TMAO	3	**0.032**	0.210
Glutarylcarnitine	+0.35	**0.024**	0.417	*Glycerophospholipid metabolism:*<br>Glycerophosphocholine, Choline	3	0.071	0.407
Adenosine Monophosphate "AMP"	-0.71	**0.028**	0.433	*Biosynthesis of alkaloids derived from histidine, purine:*<br>AMP, Caffeine, Theophylline, IMP	9	0.096	0.408
Retinol	-0.49	**0.040**	0.483	*Biosynthesis of plant secondary metabolites:*<br>AMP, Caffeine, Theophylline, IMP	10	0.097	0.408
Caffeine	-0.54	**0.043**	0.483				
Theophylline	-0.54	**0.043**	0.483				
Maleic Acid	-0.44	**0.046**	0.483				
Urate	-0.49	0.056	0.552				
2-Hydroxyisobutyrate/2-Hydroxybutyrate	-0.54	0.062	0.569				
Histidine	+0.46	0.073	0.571				
Inosine Monophosphate "IMP"	-0.20	0.078	0.571				
isoValerylcarnitine	-0.39	0.083	0.571				
Choline	-0.35	0.095	0.571				
Hydroxyproline	-0.26	0.095	0.571				
Reduced Glutathione	-0.23	0.097	0.571				

Mann Whitney U-test for metabolite abundance and globaltest for pathway alterations (limited to unadjusted P<0.1). Individual metabolites with differential abundance between weight-losing (>5% weight loss over the prior six months) and weight-stable males with cancer are listed with their corresponding pathways.

$Log_2(FC) = Log_2$(mean expression in the weight loss group) - Log_2(mean expression in the weight stable group); negative indicates lower expression in the weight loss group. Adj., adjusted; Det., detected.

Supplemental Table 2. Metabolomic Alterations in Muscle and Plasma Associated with Low Muscularity in Cancer

Correlations	r	P-value	Adj. P-value	globaltest	# Det.	P-value	Adj. P-value
**Plasma Metabolites**				**Plasma Pathways**			
2-Aminoadipate (160.0/116.0)	0.50	**<0.001**	**0.045**	_Lysine degradation_: 2-Aminoadipate, Lysine, 5-Aminovaleric Acid, Cadaverine, Carnitine	10	**0.003**	0.109
Uridine (245.2/113.1)	0.47	**0.001**	**0.045**	_ABC Transporters_: Uridine, Carnitine	9	**0.005**	0.109
Valine (118.0/72.0)	0.47	**0.001**	**0.045**	_Phenylalanine, tyrosine, tryptophan biosynthesis_: Phenylalanine, Indole, Tryptophan, PPA	4	**0.007**	0.109
Homoarginine (189.1/144.0)	0.47	**0.001**	**0.045**	_Tyrosine metabolism_: Tyrosine, Epinephrine	4	**0.013**	0.126
Lysine (147.0/84.0 (2))	0.43	**0.003**	0.104	_Alanine, aspartate, glutamate metabolism_: gamma-Aminobutyrate, Alanine	9	**0.013**	0.126
Tyrosine (182.1/136.0)	0.41	**0.005**	0.119	_Butanoate metabolism_: gamma-Aminobutyrate	4	**0.017**	0.126
gamma-Aminobutyrate (102.0/84.0)	0.41	**0.006**	0.119	_Pyrimidine metabolism_: Uridine	7	**0.020**	0.126
Glutamic acid (148.0/84.0)	0.40	**0.007**	0.119	_Branched chain amino acid biosynthesis_: Valine, Leucine/D-Norleucine, iso-Leucine/allo-isoLeucine, Pyruvate	4	**0.022**	0.126
Leucine/D-Norleucine (132.0/86.0)	0.39	**0.007**	0.119	_beta-Alanine metabolism_: gamma-Aminobutyrate, Histidine	6	**0.023**	0.126
isoValerylcarnitine (246.2/85.0)	0.39	**0.008**	0.119	_Caffeine metabolism_: Xanthine, Theophylline, Caffeine	4	**0.028**	0.136
Xanthine (151.0/108.0)	0.38	**0.010**	0.140	_Biosynthesis of alkaloids derived from histidine, purine_: Xanthine, Theophylline, Caffeine, Hypoxanthine	6	**0.030**	0.136
Urate (167.0/124.0)	0.36	**0.014**	0.180	_Monobactam biosynthesis_: Tyrosine	4	**0.039**	0.152
Alanine (90.0/44.0)	0.36	**0.016**	0.187	_Tryptophan metabolism_: Indole, Tryptophan	6	**0.040**	0.152
Indole (118.0/91.0)	0.35	**0.018**	0.189	_Arginine, proline metabolism_: 5-Aminovaleric Acid, gamma-Aminobutyrate	13	**0.042**	0.152
Cholecalciferol (385.2/91.0)	0.35	**0.018**	0.189	_Taurine, hypotaurine metabolism_: Alanine	3	**0.046**	0.155
Pyruvate (87.0/43.0)	0.34	**0.021**	0.192	_Cysteine, methionine metabolism_: Alanine, Cysteine-S-Sulfate	9	0.051	0.159
Cysteine-S-Sulfate (200.0/136.0)	-0.34	**0.023**	0.192	_Bile secretion_: 1-Methylnicotinamide, Urate	4	0.054	0.159
Tryptophan (205.1/146.0)	0.34	**0.024**	0.192	_Glycolysis, gluconeogenesis_: Pyruvate	4	0.072	0.192

Phenylalanine (166.0/120.0)	0.34	**0.024**	0.192	*Phenylalanine metabolism:* Phenylalanine, PPA	6	0.075	0.192
Ethanolamine (62.0/44.0)	0.33	**0.028**	0.211	*Glycerophospholipid metabolism:* Ethanolamine	4	0.077	0.192
iso-Leucine/allo-isoLeucine (132.0/86.0 (2))	0.33	**0.029**	0.211	*Biosynthesis of plant secondary metabolites:* Xanthine, Theophylline, PPA, Caffeine	11	0.091	0.216
N-AcetylGlycine (116.0/74.0)	-0.32	**0.031**	0.211				
S-Methylcysteine (136.0/119.0)	0.32	**0.032**	0.211				
1-Methylnicotinamide (137.0/94.0)	0.29	0.056	0.359				
Cadaverine (103.0/86.0)	0.28	0.059	0.361				
Theophylline (179.1/164.0)	0.28	0.067	0.382				
Epinephrine (184.1/166.0)	0.27	0.069	0.382				
Glucose-6-Phosphate “G6P” (259.1/97.0)	0.27	0.069	0.382				
Pyroglutamic Acid (130.0/84.0)	-0.26	0.081	0.382				
Glyceraldehyde (89.0/59.0)	0.26	0.081	0.382				
Phenylpropionic Acid “PPA” (163.0/91.0)	0.26	0.081	0.382				
1-Methyladenosine (282.2/150.1)	0.26	0.081	0.382				
Caffeine (195.1/138.0)	0.26	0.082	0.382				
Hypoxanthine (135.0/92.0)	0.26	0.087	0.393				
Carnitine (162.0/85.0)	0.26	0.090	0.393				
Histidine (156.0/110.0)	0.25	0.092	0.393				
5-Aminovaleric Acid (118.0/101.0)	0.25	0.099	0.399				
Uridine Diphosphate N-Acetylglucosamine “UDP-GlcNAc” (606.2/385.0)	0.25	0.099	0.399				
**Skeletal Muscle Metabolites**				**Skeletal Muscle Pathways**			
Glycerophosphocholine	0.68	**<0.001**	**<0.001**	*Fatty acid metabolism:* Retinol, Succinylcarnitine, isoValerylcarnitine, 3HBA	5	**0.002**	**0.016**
Carnosine	0.52	**<0.001**	**0.025**	*Lipid metabolism:* Retinol, Succinylcarnitine, isoValerylcarnitine, 3HBA	5	**0.002**	**0.016**
Anserine	0.51	**0.001**	**0.025**	*Lipid peroxidation:* Retinol, Succinylcarnitine, isoValerylcarnitine, 3HBA	5	**0.002**	**0.016**
Inositol	0.49	**0.001**	**0.031**	*Lipid transport:* Retinol, Succinylcarnitine, isoValerylcarnitine, 3HBA	5	**0.002**	**0.016**
Reduced Glutathione	0.44	**0.003**	**0.070**	*Cell signaling:* Retinol, isoValerylcarnitine	3	**0.002**	**0.016**
Deoxycytidine Monophosphate “DCMP”	0.44	**0.003**	**0.070**	*Bile secretion:* Reduced Glutathione, 1-Methylnicotinamide	3	**0.003**	**0.022**
N-AcetylGlycine	-0.42	**0.005**	**0.095**	*Galactose metabolism:*	5	**0.011**	**0.070**

				Inositol			
1-Methylnicotinamide	-0.38	**0.012**	0.190	*Histidine metabolism*:<br>Carnosine, Anserine	5	**0.015**	**0.085**
Niacinamide	0.37	**0.013**	0.190	*Glycerophospholipid metabolism*:<br>Glycerophosphocholine	3	**0.017**	**0.085**
isoValerylcarnitine	0.37	**0.014**	0.190	*Nicotinate, nicotinamide metabolism*:<br>1-Methylnicotinamide, Niacinamide	5	**0.026**	0.118
3-Hydroxybutyric Acid "3HBA"	-0.33	**0.030**	0.365	*beta-Alanine metabolism*:<br>Carnosine, Anserine	7	0.062	0.258
Retinol	-0.33	**0.032**	0.365				
Valine	0.31	**0.044**	0.404				
Glucose-6-Phosphate "G6P"	0.31	**0.045**	0.404				
Succinylcarnitine	0.30	**0.048**	0.404				
Phosphocreatine	0.30	**0.049**	0.404				
Allantoin	-0.30	**0.050**	0.404				
Glucosamine-6-Phosphate	0.29	0.060	0.454				
G1P/F1P/F6P (sugar phosphates)	0.28	0.071	0.484				
Homoarginine	0.28	0.073	0.484				
Inosine Monophosphate "IMP"	0.28	0.074	0.484				
Lysine	0.27	0.084	0.518				
Creatinine	0.26	0.093	0.518				
Adenosine	-0.26	0.095	0.518				

Spearman correlation coefficients (r) between metabolite abundance and lumbar skeletal muscle cross-sectional area (limited to unadjusted P<0.1) and globaltest for pathway alterations with low muscularity in men with cancer. Individual metabolites that were associated with muscle cross-sectional area are listed with their corresponding pathways, or "n/a" if no metabolites listed are associated with that pathway. Adj., adjusted; Det., detected.

Supplemental Table 3. Metabolomic Alterations in Muscle and Plasma Associated with Worse Physical Function in Cancer

Correlations	r	P-value	Adj. P-value	globaltest	# Det.	P-value	Adj. P-value
**HGS: Plasma Metabolites**				**HGS: Plasma Pathways**			
Indole-3-Propionate (188.0/59.0)	0.45	**0.001**	**0.088**	_Branched chain amino acid biosynthesis_: Threonine	4	**0.018**	0.730
Ergocalciferol (397.2/91.0)	0.39	**0.004**	0.274	_Lysine degradation_: Pipecolate, Deoxycarnitine, 5-Aminovaleric Acid	10	0.056	0.730
Theophylline (179.1/164.0)	0.37	**0.005**	0.274	_Monobactam biosynthesis_: Threonine	4	0.067	0.730
Threonine (120.0/74.0)	0.36	**0.007**	0.274	_Glycine, serine, threonine metabolism_: Threonine, Glycine, Guanidinoacetate, Dimethylglycine	11	0.071	0.730
Pipecolate (130.0/84.0 (2))	0.34	**0.012**	0.374	_Aminobenzoate degradation_: n/a	3	0.073	0.730
Deoxycarnitine (147.0/87.0)	0.33	**0.015**	0.392	_Arginine, proline metabolism_: 4-Guanidinobutanoate, Guanidinoacetate, 5-Aminovaleric Acid	13	0.096	0.798
Caffeine (195.1/138.0)	0.28	**0.040**	0.809				
Glycine (76.0/30.0)	0.28	**0.042**	0.809				
4-Guanidinobutanoate (146.0/87.0)	0.27	**0.048**	0.818				
Melatonin (231.1/172.0)	0.25	0.063	0.844				
Glucoronate (193.0/73.0)	-0.25	0.064	0.844				
Dimethylglycine (104.0/58.0)	0.25	0.069	0.844				
Guanidinoacetate (116.0/74.0 (2))	0.25	0.071	0.844				
5-Aminovaleric Acid (118.0/101.0)	0.24	0.079	0.869				
7-Methylguanine (166.0/149.0)	0.23	0.089	0.884				
**HGS: Skeletal Muscle Metabolites**				**HGS: Skeletal Muscle Pathways**			
5'-Methylthioadenosine	-0.30	**0.032**	0.982	_Glutathione metabolism_: Oxidized Glutathione, Cadaverine	4	**0.045**	0.953
Oxidized Glutathione	0.29	**0.034**	0.982	_Oxidative phosphorylation_: Succinate, ADP, NAD	4	0.072	0.953
gamma-Aminobutyrate	-0.29	**0.036**	0.982				
Succinate	-0.28	**0.041**	0.982				
Lactose/Trehalose	0.27	0.057	0.982				
Glutamic acid	-0.26	0.061	0.982				
Urate	-0.26	0.067	0.982				
Adenosine Diphosphate “ADP”	-0.25	0.075	0.982				

Nicotinamide Adenine Dinucleotide “NAD”	-0.25	0.076	0.982				
Cadaverine	-0.24	0.082	0.982				
Sucrose	-0.24	0.085	0.982				
Pipecolate	0.24	0.093	0.982				
**SCP: Plasma Metabolites**				**SCP: Plasma Pathways**			
Cadaverine (103.0/86.0)	0.48	**0.019**	0.992	n/a			
Fructose (179.0/89.0 (3))	-0.44	**0.033**	0.992				
Succinate (117.0/73.0)	-0.41	**0.048**	0.992				
3-Indoxyl Sulfate (212.0/80.0)	-0.35	0.090	0.992				
Pyroglutamic Acid (130.0/84.0)	-0.35	0.098	0.992				
**SCP: Skeletal Muscle Metabolites**				**SCP: Skeletal Muscle Pathways**			
Guanidinoacetate	0.57	**0.005**	0.312	*Amino sugar, nucleotide sugar metabolism*: Glucosamine-6-Phosphate, UDP-GlcNAc	3	**0.018**	0.545
Glycine	0.55	**0.006**	0.312	*Phenylalanine, tyrosine, tryptophan biosynthesis*: Phenylalanine	3	**0.045**	0.545
Carnosine	0.54	**0.008**	0.312	*Glycolysis, gluconeogenesis*: n/a	4	0.067	0.545
Anserine	0.52	**0.010**	0.312	*Histidine metabolism*: Carnosine, Anserine, Histamine	5	0.067	0.545
Phenylalanine	0.50	**0.013**	0.312	*Glycine, serine, threonine metabolism*: Guanidinoacetate, Glycine	11	0.072	0.545
Leucine/D-Norleucine	0.50	**0.014**	0.312	*Fructose, mannose metabolism*: n/a	4	0.081	0.545
iso-Leucine/allo-isoLeucine	0.45	**0.029**	0.566	*Cysteine, methionine metabolism*: Methionine, Methionine Sulfoxide	9	0.098	0.545
Histamine	0.44	**0.034**	0.566				
Glucosamine-6-Phosphate	0.43	**0.039**	0.566				
Uridine Diphosphate N-Acetylglucosamine “UDP-GlcNAc”	0.42	**0.044**	0.566				
Methionine	0.41	**0.045**	0.566				
betaAlanine	0.38	0.071	0.698				
Niacinamide	0.38	0.071	0.698				
Guanosine	0.38	0.071	0.698				
Methionine Sulfoxide	0.35	0.092	0.838				

Spearman correlation coefficients (r) between metabolite abundance and physical function (limited to unadjusted $P<0.1$) and globaltest for pathway alterations with handgrip strength (HGS) or stair climb power (SCP) in men with cancer. Individual metabolites that were associated with HGS or SCP are listed with their corresponding pathways, or “n/a” if no metabolites listed are associated with that pathway. Adj., adjusted; Det., detected.
