# Supplementary material for: Distinct Metabolomic Alterations Are Associated With Physical Function, Weight Loss, and Muscle Mass in Men With Cancer
Source: J Cachexia Sarcopenia Muscle. 2026 Jan 18;17(1):e70183. doi: 10.1002/jcsm.70183 (PMC12813416; doi:10.1002/jcsm.70183)
Supplement: Supplementary file 4 — Table S4: Nominal Spearman's correlations between plasma metabolite abundances and skeletal muscle index. Table S5: Nominal Spearman's correlations between muscle metabolite abundances and skeletal muscle index. [file JCSM-17-e70183-s001.pdf]

Supplemental Table 4. Nominal Spearman's correlations between plasma metabolite abundances and skeletal muscle index

**Plasma Metabolite**	***r***	***P***	**FDR**
Homoarginine (189.1 / 144.0)	0.530	0.000	0.034
Uridine (245.2 / 113.1)	0.444	0.002	0.156
2-Aminoadipate (160.0 / 116.0)	0.435	0.003	0.156
Valine (118.0 / 72.0)	0.373	0.012	0.324
Xanthine (151.0 / 108.0)	0.355	0.017	0.324
iso-Leucine /allo-isoLeucine (132.0 / 86.0 (2))	0.350	0.019	0.324
Tyrosine (182.1 / 136.0)	0.345	0.021	0.324
isoValerylcarnitine (246.2 / 85.0)	0.344	0.021	0.324
Lysine (147.0 / 84.0 (2))	0.343	0.022	0.324
Leucine /D-Norleucine (132.0 / 86.0)	0.338	0.024	0.324
Indole (118.0 / 91.0)	0.327	0.029	0.324
Citrulline (174.0 / 131.0)	-0.323	0.031	0.324
Cholecalciferol (385.2 / 91.0)	0.323	0.031	0.324
Urate (167.0 / 124.0)	0.321	0.032	0.324
Epinephrine (184.1 / 166.0)	0.321	0.032	0.324
2-Hydroxyisobutyrate/2-Hydroxybutyrate (103.0 / 57.0)	0.317	0.034	0.324
Hypoxanthine (135.0 / 92.0)	0.314	0.036	0.324
Glyceraldehyde (89.0 / 59.0)	0.311	0.038	0.324
Glutamic acid (148.0 / 84.0)	0.303	0.043	0.337
1-Methyladenosine (282.2 / 150.1)	0.303	0.044	0.337
Alanine (90.0 / 44.0)	0.300	0.046	0.337

Supplemental Table 5. Nominal Spearman's correlations between muscle metabolite abundances and skeletal muscle index

**Muscle Metabolite**	***r***	***P***	**FDR**
Glycerophosphocholine	0.692	0.000	0.000
Anserine	0.512	0.001	0.025
Carnosine	0.512	0.001	0.025
Inositol	0.482	0.001	0.042
Reduced Glutathione	0.461	0.002	0.049
DCMP	0.460	0.002	0.049
N-AcetylGlycine	-0.437	0.004	0.071
Allantoin	-0.430	0.004	0.073
3HBA	-0.387	0.011	0.154
1-Methylnicotinamide	-0.380	0.012	0.154
isoValerylcarnitine	0.380	0.012	0.154
Homoarginine	0.338	0.027	0.308
Aspartic Acid	-0.316	0.039	0.405
Retinol	-0.313	0.041	0.405
